# The MHC Class II transactivator CIITA inhibits Tax-1-mediated HTLV-1 expression and NF-kB activation

**DOI:** 10.1186/1742-4690-11-S1-P64

**Published:** 2014-01-07

**Authors:** Rawan Abdallah, Greta Forlani, Roberto S Accolla, Giovanna Tosi

**Affiliations:** 1 Department of Surgical and Morphological Sciences, University of Insubria, Varese, Italy

## 

Human T-cell Lymphotropic Virus type-1 (HTLV-1) is the causative agent of an aggressive malignancy of CD4+ T lymphocytes. Many evidences have shown that constitutive activation of NF-kB pathway by Tax-1 is crucial for T-cell transformation. We previously demonstrated that CIITA, the master regulator of MHC class II gene transcription, inhibits HTLV-1 replication by blocking the transcription function of the viral transactivator Tax-1. Here we show that CIITA suppresses also Tax-1-mediated activation of the NF-kB pathway. Of note, CIITA interacts with and retains Tax-1 in cytoplasmic fraction and inhibits Tax-1-dependent nuclear translocation of RelA. Moreover, the overexpression of CIITA does not affect Tax-1 interaction with both RelA and IKKg. CIITA acts by suppressing at least the canonical NF-kB pathway, in that it also inhibits activation of NF-kB by Tax-2, which is known to activate NF-kB through the canonical but not the non-canonical pathway. Future studies will be focused to identify the fine mechanisms through which CIITA exerts this inhibitory function. Overall, these findings indicate that CIITA, beside acting as viral restriction factor against HTLV-1 infection, might counteract Tax-1 transforming activity. Thus, assessing the molecular basis of CIITA-mediated Tax-1 inhibition may be important in defining new strategies to control HTLV-1 spreading and oncogenic potential. Supported by: Fondazione Cariplo (2008-2230), AIRC (IG 8862) and Miur PRIN-COFIN 2008. Rawan Abdallah PhD Fellowship is granted by Fondazione Cariplo Bando 2009: Promuovere la formazione di capitale umano di eccellenza: Percorso di eccellenza in tecnologie biomedicali innovative.

